# Adjuvant chemotherapy in patients with operable granulosa cell tumors of the ovary: a surveillance, epidemiology, and end results cohort study

**DOI:** 10.1002/cam4.1447

**Published:** 2018-04-17

**Authors:** Anton Oseledchyk, Renee L. Gennarelli, Mario M. Leitao, Carol A. Aghajanian, Alexia Iasonos, Oliver Zivanovic, Dmitriy Zamarin

**Affiliations:** ^1^ Department of Radiology Memorial Sloan Kettering Cancer Center New York New York 10065; ^2^ Department of Epidemiology and Biostatistics Memorial Sloan Kettering Cancer Center New York New York 10065; ^3^ Gynecology Service Department of Surgery Memorial Sloan‐Kettering Cancer Center New York New York 10065; ^4^ Weill Cornell Medical College New York New York 10065; ^5^ Gynecologic Medical Oncology Service Department of Medicine Memorial Sloan‐Kettering Cancer Center New York New York 10065

**Keywords:** Chemotherapy, end results, epidemiology, granulosa cell tumor, ovary, surveillance

## Abstract

Adjuvant chemotherapy is recommended for patients with resected high‐risk adult granulosa cell tumors (GCT), although strong data to support this are lacking. The objective of this study was to assess the outcomes of GCT patients, with the specific focus on patients that received adjuvant chemotherapy with curative intent (stage I‐III), reported in a large national cancer registry. Data from the Surveillance, Epidemiology, and End Results (SEER) database between 2000 and 2013 were used for analysis. Patient and disease characteristics were extracted and analyzed for association with administration of chemotherapy. Impact on disease‐specific survival (DSS) was analyzed using log‐rank test. A total of 739 patients with surgically treated adult GCT were identified. Median age was 51 years. 570 (77%) patients were stage I, 87 (12%) were stage II, and 82 (11%) were stage III. Adjuvant chemotherapy was administered to 176 (24%) patients. Young age, higher stage, and hysterectomy were associated with chemotherapy administration. Higher disease stage was associated with decreased five‐year DSS (IA/B 98.5%, IC 95.1%, II 86.1%, III 83.5%, *P* < 0.01). Notably, administration of adjuvant chemotherapy was not associated with improved five‐year DSS (*P* = 0.45) regardless of disease stage (stage IA/B: 96% with chemotherapy vs. 99% without chemotherapy; *P* = 0.64), (stage IC: 97% with chemotherapy vs. 94% without chemotherapy; *P* = 0.49), (stage II: 89% with chemotherapy vs. 83% without chemotherapy; *P* = 0.56), (stage III: 73% with chemotherapy vs. 93% without chemotherapy; *P* = 0.18). In this analysis, chemotherapy was not found to be associated with improved DSS of patients with operable disease regardless of stage, questioning the role for adjuvant chemotherapy in GCT.

## Introduction

Granulosa cell tumors (GCTs) of the ovary are the most common type of malignant ovarian sex cord‐stromal tumors, albeit they comprise only 2–5% of all ovarian cancers 1. Typically, patients present at an early stage with large unilateral tumors. Although GCT has an excellent prognosis, its indolent behavior results in late recurrences, with nearly half of the relapses occurring more than 5 years after primary diagnosis 2. This requires extremely long follow‐up times to generate meaningful clinical data and raises the question of whether aggressive adjuvant chemotherapy is beneficial in GCT.

The National Comprehensive Cancer Network (NCCN) Guidelines recommend adjuvant chemotherapy for advanced stage as well as high‐risk FIGO stage I disease, which is based on factors like intraoperative rupture or poor differentiation 3. Predominantly, adjuvant chemotherapy with bleomycin, etoposide, and cisplatin (BEP) is used. The efficacy of this regimen is currently being compared to a combination of Carboplatin and Paclitaxel in the GOG‐0264 (NCT01042522) study. While this might alleviate the morbidity of adjuvant treatment, it will not answer the question of whether there is rationale for an adjuvant chemotherapeutic treatment in GCT altogether, unless one treatment arm demonstrates significant improvement in survival over another. Unfortunately, prospective studies comparing adjuvant chemotherapy to observation alone have not been performed and will most likely not be feasible in the future. Therefore, current recommendations are mainly based on small retrospective analyses and on extrapolations of data from the more common epithelial ovarian cancer subtypes.

The inherent weaknesses of retrospective analyses of this rare cancer entity resulted in contradicting data and a highly variable practice nationwide. While some studies suggest benefit 4, 5, 6, others show no survival advantage in patients treated with adjuvant chemotherapy 7, 8, 9, 10, 11, 12, 13. Additionally, most of these studies suffer from small patient numbers with very low rates of chemotherapy administration in early stage GCT, by that complicating the interpretation further. A recent study by Seagle et al. 13 used the National Cancer Database to analyze the outcomes of women with stage II–IV ovarian GCT. Interestingly, in a matched cohort analysis of patients that received or did not receive adjuvant chemotherapy for stages II–IV disease, no significant differences in overall survival were found with chemotherapy administration. As GCTs tend to exhibit a relatively slow disease course, the overall survival might not be an accurate representation of disease course. As such, in the current study, we sought to examine disease‐specific survival (DSS) in patients with GCT that received or did not receive adjuvant chemotherapy and to evaluate the association of chemotherapy with DSS in the different disease stages using the SEER database. As stage IV disease has a strong indication for systemic chemotherapy, the major goal of the current study was to examine the association between chemotherapy administration and outcomes in patients with earlier disease stages.

## Methods

### Data source

Data were extracted from the Surveillance, Epidemiology, and End Results (SEER) database. SEER is a population‐based cancer registry maintained by the National Cancer Institute. The database “SEER 18 Regs Custom Data with chemotherapy recode, Nov 2015 (2000–2013) <Katrina/Rita Population Adjustment>” was used 14. From 81,287 patients with any diagnosis of ovarian origin defined by the variable “Site and Morphology – Primary Site = ‘C56.9‐Ovary’” the following histologies have been extracted: 8620/3 Granulosa cell tumor, malignant (*n* = 1024); 8621/3 Granulosa cell‐theca cell tumor, malignant (*n* = 1); 8622/3 Juvenile granulosa cell tumor, malignant (*n* = 17). For further analysis, juvenile granulosa cell tumors were excluded due to low patient numbers.

### Study population

**Figure 1:**
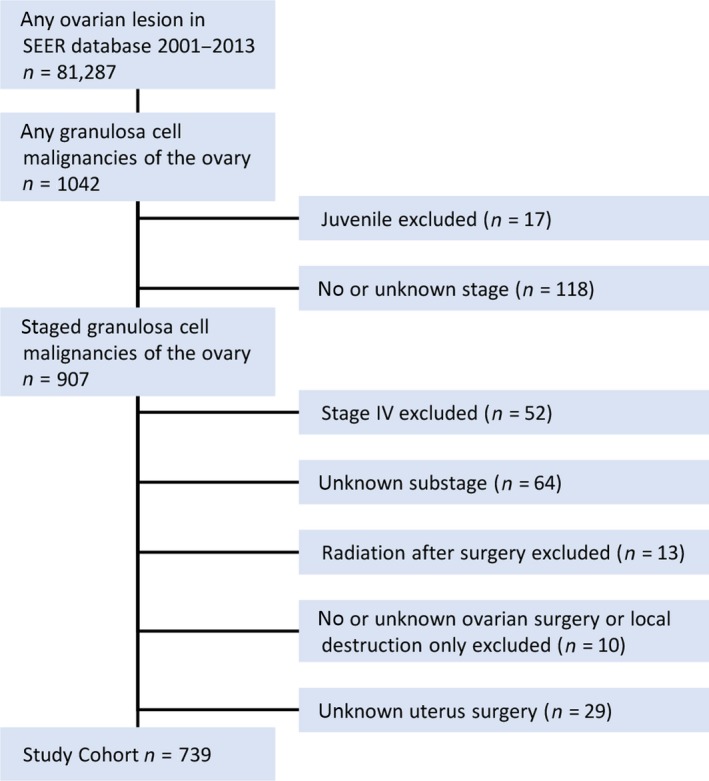
CONSORT diagram of patient selection from SEER database.

The following variables were exported from SEER*Stat 8.3.2 software to Microsoft Excel for further analysis: age at diagnosis, race, stage, chemotherapy, radiation therapy, surgery for primary site, number of lymph nodes dissected, months of follow–up, and disease‐specific survival (DSS). DSS was defined as time from date of primary diagnosis to date of cancer‐related death. Patients with stage IV (*n* = 52), unknown stage (*n* = 118) or unknown substage (*n* = 64) were excluded from the analysis. Because of low number of patients undergoing adjuvant radiation therapy (*n* = 13), those cases were excluded. Similarly, patients that did not undergo a resection of the tumor (*n* = 10) or had no documentation regarding uterus surgery (*n* = 29) were excluded. The final study cohort of 739 patients was subjected to further analyses (Fig. 1). DSS outcomes were available for all patients, and all patients were included in analyses of association of chemotherapy with outcomes.

Administration of chemotherapy was reported either as “yes” or “no/unknown” in the extracted dataset. Prior analysis comparing the variables for treatment of the SEER registry with Medicare claims reported a positive predictive value of 94.7% for the chemotherapy variable in ovarian cancer with sensitivity of 84.4%, suggesting that approximately 15% of the patients in the “no/unknown” group in the current study may have also received chemotherapy 15. In addition, SEER does not distinguish between adjuvant vs. neoadjuvant chemotherapy. As neoadjuvant chemotherapy is not a standard treatment approach for stages I–III GCT, the analyses were performed with an assumption that the vast majority of the patients received chemotherapy in the postoperative setting. Additional characteristics known to potentially influence the outcomes of patients, such as comorbidities, performance status, surgery details, residual disease, and time to chemotherapy initiation, were not available; thus, the presence of additional confounding variables could not be ruled out.

### Statistical analysis

There were no cases with missing data. *χ* ^2^ test was used to compare disease and patient characteristics between treatment groups. Predictors of the use of chemotherapy were determined by binomial logistic regression. Survival analyses were performed using the Kaplan–Meier method log‐rank tests. Despite the known limitation of this methodology for survival analyses, multivariate cox proportional hazards regression analysis could not be used to examine the effects of chemotherapy on survival as chemotherapy start dates were not known and receipt of adjuvant chemotherapy is a time‐dependent covariate that has to be analyzed as such. Due to potential misclassification of receipt of chemotherapy, we conducted sensitivity analyses to explore the impact that misclassification of chemotherapy receipt could have on survival estimates. Based on the previously reported sensitivity of 84.4% for the SEER chemotherapy variable in ovarian cancer 15, we estimated that about 15% (*n* = 85) of the patients in the “no/unknown” chemotherapy group may have been misclassified and would have actually received chemotherapy. To account for this, we ran 1000 simulations that randomly re‐classified 15% of the patients in the “no/unknown” group as “yes” and compared survival using the log‐rank test. A two‐tailed *P* value of ≤0.05 was regarded as statistically significant. All statistical analyses were performed with IBM SPSS 24.0.0.0 (IBM Corp., Armonk, NY) or SAS 9.4 (SAS Institute Inc., Cary, NC).

## Results

### Patients demographics

A total of 739 patients with surgically resected adult granulosa cell tumor (GCT) of known FIGO stages I–III disease were identified (Table 1). The median age at diagnosis was 51 years, with 250 patients (34%) being younger than 46 years of age. The majority of patients were white (*n* = 513, 70%) and a quarter were black (*n* = 176, 24%). A total of 570 (77%) patients were FIGO stage I, 87 (12%) were FIGO stage II, and 82 (11%) were FIGO stage III. There were only eight patients of substage IB; therefore, they were grouped with patients from substage IA into substage IA/B (*n* = 439; 58%). As patient numbers in the distinct FIGO stage II and III substages were ranging between 14 and 53 patients, the substages were merged and analyzed as FIGO stage II and FIGO stage III.

**Table 1:** Patient demographics of the selected study cohort

Patient characteristics		
	*n*	%
Age at diagnosis		
Median	51	
Range	6‐93	
>45	489	66.2
≤45	250	33.8
Race		
Asian	39	5.3
Black	176	23.8
Native	3	0.4
Unknown	8	1.1
White	513	69.4
Stage		
IA	423	57.2
IB	8	1.1
IC	139	18.8
IIA	14	1.9
IIB	53	7.2
IIC	20	2.7
IIIA	21	2.8
IIIB	23	3.1
IIIC	38	5.1
Stage combined		
IA/B	431	58.3
IC	139	18.8
II	87	11.8
III	82	11.1
Chemotherapy		
No/Unknown	563	76.2
Yes	176	23.8
Ovarian surgery		
BSO	235	31.8
Debulking	58	7.8
Ovary only	27	3.7
BSO or USO	288	39.0
USO	131	17.7
Hysterectomy		
None	216	29.2
Yes	523	70.8
Lymph nodes dissected		
>10	175	23.7
1–10	218	29.5
Unknown	19	2.6
None	327	44.2

Surgical resection consisted of unilateral resection of the ovary and tumor in 27 (3.7%) patients or unilateral salpingo‐oophorectomy (USO) in 131 (18%) patients, a bilateral salpingo‐oophorectomy (BSO) in 235 (32%) patients or an extensive debulking procedure in 58 (7.8%) patients. In 288 (40%) patients, it remained unknown whether USO or BSO was performed; therefore, due to limited interpretability, this variable was excluded from further statistical analyses. Instead, hysterectomy was used as a surrogate for completeness of primary surgery versus an incomplete fertility sparing procedure. A total of 523 (70%) patients had their uterus removed at primary surgery. Dissection of lymph nodes was performed in 412 (56%) of the patients with 175 (24%) patients having more than 10 and 218 (30%) between 1 and 10 lymph nodes removed. For 19 (2.6%) patients, lymph node dissection was documented but number of resected lymph nodes was not. However, as lymph node dissection is not recommended for the primary staging of GCT 3, the presence or absence of lymph node dissection was not taken into account in further statistical analyses.

### Predictors for receiving adjuvant chemotherapy

Adjuvant chemotherapy was administered in 176 (24%) patients (Table 1). Patients undergoing chemotherapy were significantly different from patients who did not (Table 2). Patients receiving adjuvant chemotherapy were younger (median age 48 vs. 52; *P* = 0.003) and more likely to be in a reproductive age defined as younger than 46 years old (40% vs. 32%; *P* = 0.016). Also, patients with higher stage (*P* < 0.001) were more likely to undergo chemotherapy. Binomial regression analysis (Table 3) confirmed young age (OR: 0.97; 95%CI: 0.95–0.98; *P* < 0.001) and higher stage (IC vs. IA OR: 5.32; 95%CI: 3.20–8.86; *P* < 0.001; II vs. IA OR: 18.63; 95%CI: 10.47–33.17; *P* < 0.001; III vs. IA OR: 15.25; 95%CI: 8.58–27.10; *P* < 0.001) as well as removal of the uterus (OR 1.67, 95%CI: 1.04–2.69, *P* = 0.034) to be independently associated with chemotherapy administration.

**Table 2:** Association between patient characteristics and adjuvant chemotherapy

Association with chemotherapy administration					
	No chemotherapy		Chemotherapy		
	*n*	%	*n*	%	
Age					
Median	52		48		**0.003** a
Range	6–93		15–83		
Age at diagnosis					
0–29	31	5.5	15	8.5	**0.016**
30–39	82	14.6	28	15.9	
40–49	125	22.2	53	30.1	
50–59	169	30.0	48	27.3	
60–69	67	11.9	20	11.4	
70+	89	15.8	12	6.8	
Age binary					
>45	384	68.2	105	59.7	**0.023**
≤45	179	31.8	71	40.3	
Race					
Asian	35	6.2	4	2.3	0.360
Black	134	23.8	42	23.9	
Native	2	0.4	1	0.6	
Unknown	6	1.1	2	1.1	
White	386	68.6	127	72.2	
Stage					
IA/B	395	70.2	36	20.5	**<0.001**
IC	96	17.1	43	24.4	
II	36	6.4	51	29.0	
III	36	6.4	46	26.1	
Hysterectomy					
None	173	30.7	43	24.4	0.065
Yes	390	69.3	133	75.6	

Statistical significance was calculated using chi‐square test; *P* ≤ 0.05 is regarded as statistically significant.

Statistically‐significant values are presented in bold.

a *P*‐values for continuous variables shown in blue were calculated using Mann–Whitney test.

**Table 3:** Predictors of the receipt of chemotherapy. Statistical significance was calculated using binomial logistic regression; *P* ≤ 0.05 is regarded as statistically significant. (OR > 1 chemotherapy administration more likely; OR < 1 observation more likely)

Binary logistic regression: predictors of the receipt of chemotherapy				
	OR	95% CI		*P*‐value
		Lower	Upper	
Age at diagnosis	0.968	0.954	0.982	**<0.001**
Year of diagnosis	1.006	0.956	1.058	0.826
Race				
White		*Reference*		0.110
Asian	0.221	0.070	0.703	**0.011**
Black	0.848	0.531	1.352	0.488
Native	1.480	0.053	41.372	0.818
Unknown	0.419	0.071	2.453	0.335
Stage				
IA/B		*Reference*		**<0.001**
IC	5.323	3.200	8.855	**<0.001**
II	18.633	10.466	33.171	**<0.001**
III	15.250	8.581	27.104	**<0.001**
Hysterectomy	1.674	1.041	2.693	**0.034**

Statistically‐significant values are presented in bold.

### Predictors of survival

**Figure 2:**
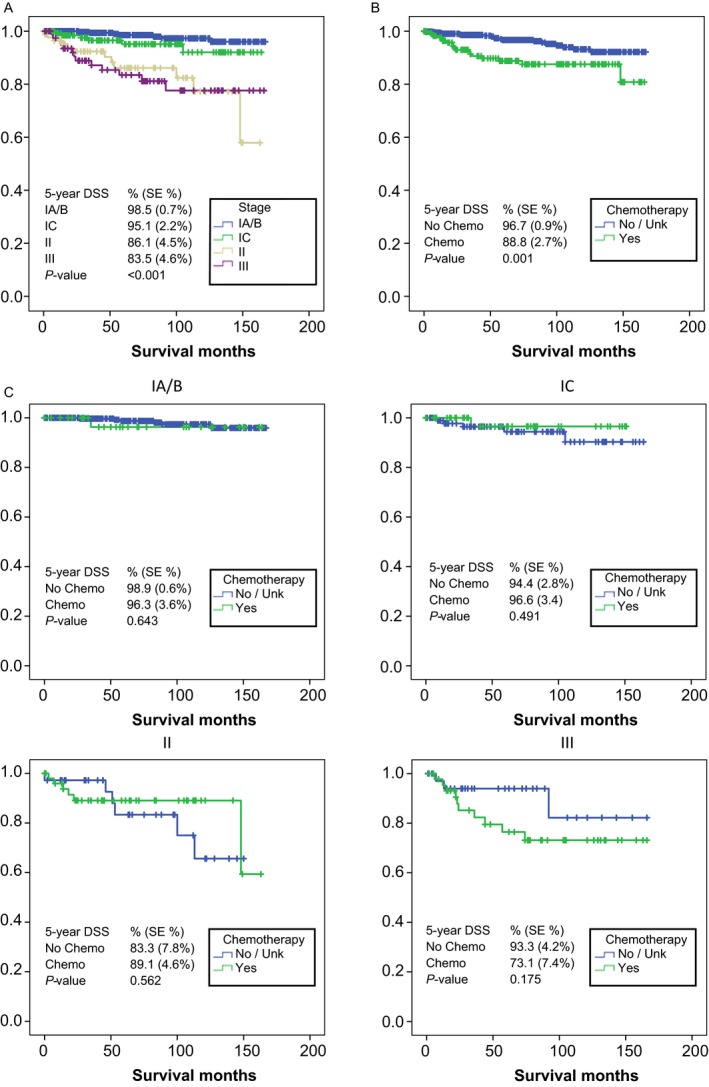
Association of chemotherapy with disease‐specific survival (DSS). Statistical significance was calculated using Kaplan–Meyer survival analysis. (A) DSS by stage/substage. (B) Association of chemotherapy with DSS in the overall cohort. (C) Association of chemotherapy with DSS in each disease stage/substage. *P* ≤ 0.05 is regarded as statistically significant.

Median follow‐up for all surviving patients was 62 months (range: 0–167 months; mean 69 months; CI95%: 65.6–72.7). Patients with substage IA/B, substage IC, stage II and stage III GCT had a five‐year DSS of 99% (SE 0.7%), 95% (SE 2.2%), 86% (SE 4.5%), and 84% (SE 4.6%), respectively (Fig. 2A). This difference was statistically significant (*P* < 0.001). Despite the fact that younger patients were more likely to receive chemotherapy, there was no statistically significant difference in DSS in patients under 45 and over 45 years of age (5yDSS: >45y 94.5% vs. <45y 95.3%, *P* = 0.406).

### Association of chemotherapy with survival

In the overall cohort, administration of chemotherapy (Fig. 2B) was associated with an impaired five‐year DSS of 89% (SE 2.7%) compared to 97% (SE 0.9%) in patients who did not receive adjuvant chemotherapy (*P* = 0.001). As the superior outcomes in the observation group were likely influenced by a larger number of stage IA/B patients compared to the chemotherapy group, the association of chemotherapy with survival was evaluated for each individual stage of GCT, whereby substages IA/B and IC were analyzed separately, given the reported higher risk of IC disease (Fig. 2C). Irrespective of stage or substage, there were no statistically significant differences in five‐year DSS between patients that received chemotherapy and those who did not (stage IA/B: 96% with chemotherapy vs. 99% without chemotherapy; *P* = 0.643), (stage IC: 97% with chemotherapy vs. 94% without chemotherapy; *P* = 0.491), (stage II: 89% with chemotherapy vs. 83% without chemotherapy; *P* = 0.562), (stage III: 73% with chemotherapy vs. 93% without chemotherapy; *P* = 0.175).

### Sensitivity analysis

After reclassifying 15% of the patients in the “no/unknown” group to “yes” for chemotherapy receipt in 1000 simulations, there was no significant difference in DSS between those with and without adjuvant chemotherapy in 11.6% of the simulations, while 88.4% of the simulations resulted in significantly lower unadjusted DSS for patients who had chemotherapy compared to those that did not. This suggests that misclassification of receipt of chemotherapy could have led to a type 1 error in the original analysis, but that it is not highly likely.

## Discussion

To date, to our knowledge, this is one of the largest reported retrospective reviews of GCT patients. We report on 431 patients with substage IA/B, 139 patients with substage IC, 87 patients with stage II and 82 patients with stage III disease. As the patients who received chemotherapy were significantly younger than the patients who did not, we focused on DSS as the primary outcome measure to avoid the confounding influence of age upon survival. We did not detect an association between chemotherapy and improved DSS in any of the subgroups or in the entire study cohort.

Despite the large patient number, this study shares the common limitations that are inherent to all SEER database analyses. First of all, the study lacks sufficient power to perform meaningful statistical analyses by substage to compare the patients that received vs. did not receive adjuvant chemotherapy due to a small number of events in all substages. Secondly, there is lack of clinically significant details that typically factor into the decision to pursue adjuvant chemotherapy, including the details of the surgery and residual disease as well as comorbidities and performance status. Third, there is a lack of information on the recurrence free survival and further treatment history that could influence the DSS outcomes. Therefore, in this dataset, we cannot account for the selection bias and have to acknowledge that the treated patients might have exhibited additional high‐risk features that we are not aware of. Lastly and importantly, multivariate cox proportional hazards regression analysis could not be applied to examine the effects of chemotherapy on DSS. Adjuvant chemotherapy is a time‐dependent variable; however, chemotherapy start dates are not captured in the SEER database. Therefore, both treatment status and outcome change over time after the date of diagnosis and for this reason cannot be analyzed by a cox regression model.

Lastly, there is a lack of information regarding the treatment regimens, the number of treatment cycles or delays, and the timing of chemotherapy initiation. The variable that was made accessible to us by the SEER database only distinguishes between “yes” as in chemotherapy administered and “no/unknown.” Noone et al. 15 compared the variables for treatment from the SEER registry with Medicare claims. The authors reported a positive predictive value of 94.7% for the chemotherapy variable in ovarian cancer. The sensitivity was 84.4%, suggesting that approximately 15% of the patients in the observation group in our study may have also received adjuvant chemotherapy. For this reason, we have performed a sensitivity analysis which simulated the reassignment of the 15% of misclassified patients to the chemotherapy cohort. In 88% of 1000 simulations, the simulated results were consistent with the original finding of chemotherapy having significantly lower DSS than no systemic treatment; therefore, a type 1 error due to misclassification is possible but unlikely.

This analysis of the SEER dataset is supportive of recent publications that questioned the value of adjuvant chemotherapy in GCT. In a previous small retrospective analysis of GCT patients in our institution's database 10, we reported on 118 patients with stage I–IV GCT. Of the 103 stage I patients, only one underwent chemotherapy. Nine of 15 stage II–IV patients received adjuvant treatment. Interestingly there was a longer recurrence free survival in the untreated patients. A recent analysis of substage IC GCT patients from the MITO‐9 study database 9 also failed to show a benefit of chemotherapy that was administered to nine of 40 patients (7 BEP and 2 carboplatin/paclitaxel). This analysis, however, used data from a very long time interval of 1965 to 2008. A qualitative Cochrane Systematic Review of GCT studies published before January 2014, encompassing a total of 535 patients similarly did not demonstrate a benefit for adjuvant chemotherapy 8. Recently, a study by Seagle et al. 13 reported on a large analysis of ovarian granulosa cell patients using the National Cancer Database. Administration of chemotherapy was not associated with improved overall survival in patients with stages II–IV disease, when compared using matched cohort analysis of 165 patients in each group 13. While supporting the previous findings by Seagle et al. using an independent dataset, this study provides additional insights into potential role of chemotherapy in different disease substages, and uses DSS as a primary outcome measure, which we believe is more meaningful in this cancer with a long natural disease history.

Current NCCN guidelines recommend the omission of adjuvant chemotherapy in stage IA/B disease, and suggest considering chemotherapy in patients with higher stage. The findings by Seagle et al. and this study, as well as other retrospective analyses suggest that adjuvant chemotherapy might also not benefit patients with higher stage disease. It does, however, appear that the extent of surgical staging may be associated with improved outcomes 13, and in fact surgery remains to be one of the most effective options for the patients with recurrent oligometastatic disease due to lack of effective systemic therapies 16, 17.

Additionally, the suggested ineffectiveness of chemotherapy outlines the necessity to develop a directed targeted therapy option for GCT. Recently, it has been shown that a mutation in FOXL2 is present in most adult GCTs 18. There are indications that FOXL2 is a driver of adult GCT pathogenesis and could be a potential target for future therapeutics, possibly in the adjuvant setting. At present, complete surgical resection remains to be the strongest predictor of outcomes in this disease 13. In view of significant short‐ and long‐term toxicities associated with the chemotherapy regimens currently used for GCT, these findings suggest that observation might be reasonable to consider even in stages II and III patients, if a complete surgical resection is achieved. These findings call for an effort to identify biomarkers predictive of benefit of adjuvant chemotherapy and to develop targeted therapies that could be more appropriate in the adjuvant setting in this disease.

## Conflict of Interest

None relevant to the current study.
